# Long non-coding RNA H19 regulates FOXM1 expression by competitively binding endogenous miR-342-3p in gallbladder cancer

**DOI:** 10.1186/s13046-016-0436-6

**Published:** 2016-10-03

**Authors:** Shou-Hua Wang, Fei Ma, Zhao-hui Tang, Xiao-Cai Wu, Qiang Cai, Ming-Di Zhang, Ming-Zhe Weng, Di Zhou, Jian-Dong Wang, Zhi-Wei Quan

**Affiliations:** 1 Department of General Surgery, Xinhua Hospital, Shanghai Jiao tong University School of Medicine, 1665 Kong Jiang Road, Shanghai, 200000 China; 2 Department of Oncology, Xinhua Hospital Affiliated to Shanghai Jiao tong University School of Medicine, Shanghai, 200092 China

**Keywords:** H19, miR-342-3p, Competing endogenous RNA, FOXM1, Gallbladder cancer

## Abstract

**Background:**

Long non-coding RNA (lncRNA) H19 has been reported to involve in many kinds of human cancers and functions as an oncogene. Our previous study found that H19 was over-expressed in gallbladder cancer (GBC) and was shown to promote tumor development in GBC. However, the competing endogenous RNA (ceRNA) regulatory network involving H19 in GBC progression has not been fully elucidated. We aim to detect the role of H19 as a ceRNA in GBC.

**Methods and Results:**

In this study, the expression of H19 and miR-342-3p were analyzed in 35 GBC tissues and matched normal tissues by using quantitative polymerase chain reaction (qRT-PCR). We demonstrated H19 was overexpressed and negatively correlated with miR-342-3p in GBC. By dual-luciferase reporter assays, RNA-binding protein immunoprecipitation (RIP) and RNA pull-down assays, we verified that H19 was identified as a direct target of miR-342-3p. QRT-PCR and Western-blotting assays demonstrated that H19 silencing down-regulated, whereas over-expression enhanced the expression of miR-342-3p targeting FOXM1 through competitively ‘sponging’ miR-342-3p. Furthermore, transwell invasion assays and cell cycle assays indicated that H19 knockdown inhibited both cells invasion and proliferation, but this effects was attenuated by co-transfection of siRNA-H19 and miR-342-3p inhibitor in GBC cells. In vivo, tumor volumes were decreased significantly in H19 silenced group compared to the control group, but was attenuated by co-transfection of shRNA-H19 and miR-342-3p inhibitor, which were stablely constructed through lenti-virus vector.

**Conclusion:**

Our results suggest a potential ceRNA regulatory network involving H19 regulates FOXM1 expression by competitively binding endogenous miR-342-3p in GBC. This mechanism may contribute to a better understanding of GBC pathogenesis and provides potential therapeutic strategy for GBC.

## Background

Gallbladder cancer (GBC) is the most common biliary tract cancer and the fifth most common gastrointestinal malignancy [1]. It is estimated that there were 10,910 new cases and 3,700 deaths from gallbladder (and other biliary) cancer in the United States in 2015 (http://www.cancer.gov/). Major advances in cancer biology had led to better understanding the mechanism of GBC tumorigenesis and then led to novel therapeutic methods [2–4]. A Phase II study on gemcitabine, oxaliplatin in combination with panitumumab, a new drug inhibiting Epidermal Growth Factor Receptor (EGFR), has shown encouraging efficacy in unresectable biliary tract and GBC [5]. However, EGFR expression ranges from 39 ~ 85 % in GBC patients, and it was not a specific marker in gallbladder marker [6, 7]. Thus, it is extreme urgency to find novel molecular target and provide potential therapeutic strategy for GBC.

Long non-coding RNAs (LncRNAs) are RNAs longer than 200 nucleotides [8]. Recently, lncRNAs had been reported to be involved in genetic and epigenetic regulation and post-transcriptional regulation [9–11]. Some lncRNAs had been reported to be functioned as prognostic marker [12, 13]. Mechanism studies about the biological role of lncRNA in caners also been proposed by some research groups [14, 15]. In GBC, HOX transcript antisense intergenic RNA (HOTAIR), metastasis-associated lung adenocarcinoma transcript 1 (MALAT1) and Colon cancer-associated transcript-1 (CCAT1) had been proved to be important for GBC progression [16–18].

LncRNA H19 is located at 11p15.5 locus, whose expression is high in fetus but decreased after birth. These favorable characteristics enable its function as a genetic biomarker [19]. Mutation of H19 in mouse zygotes causes prenatal lethality, indicating its vital role in growth and development [20]. New progress in understanding the intrinsic mechanisms of lncRNA including competitive endogenous RNA(ceRNA) paradigm [21]. MicroRNAs (miRNAs) were well-accepted regulators in cancer and other diseases [22–25]. LncRNA, such as H19, act as a molecular sponge inhibiting miRNA let-7 [26]. H19 promotes epithelial to mesenchymal transition by functioning as miRNA sponges in colorectal cancer [27]. Our previous study found H19 is upregulated in GBC, and H19 promoted the GBC cells proliferation by AKT2 [28, 29], these results further enhanced our research interests for the role H19 involving in ceRNA regulatory network in GBC progression.

In this study, we demonstrated that H19 modulated FOXM1 expression by competitively ‘sponging’ to miR-342-3p, which led to promote cells proliferation and invasion in GBC cells.

## Methods

### Patient samples

Thirty-five gallbladder carcinoma tissues and pair-matched normal gallbladder tissues in this study (collected postoperatively from January 2009 to March 2012) were obtained from patients who underwent radical resections at Xinhua Hospital (Shanghai Jiao Tong University School of Medicine, Shanghai, China) and Eastern Hepatobiliary Surgical Hospital and Institute (The Second Military University, Shanghai, China). Samples were snap-frozen in liquid nitrogen and stored at −80 °C before RNA isolation and qRT-PCR analysis. None of the patients recruited to this study received any pre-operative treatments. GBC patients were staged according to the TNM staging system (the seventh edition) of the American Joint Committee on Cancer staging system. Complete clinic-pathological follow-up data of the GBC patients were collected. The study methodology conformed to the standard set by the Declaration of Helsinki and was approved by the Human Ethics Committee of Xinhua Hospital at Shanghai Jiao Tong University (Shanghai, China). All patients had signed inform consent forms.

### Cell culture

Three human GBC cell lines (GBC-SD, EHGB-1 and NOZ) were used in this study. GBC-SD was purchased from Cell Bank of the Chinese Academy of Science (Shanghai, China). NOZ was purchased from the Health Science Research Resources Bank (Osaka, Japan). EHGB-1 was a generous gift from Eastern Hepatobiliary Surgical Hospital and Institute, The Second Military University, Shanghai, China. The cell lines were cultured in Dulbecco’s modified Eagle’s medium (Gibco BRL, Grand Island, NY, USA), containing 10 % fetal bovine serum (FBS, HyClone, Invitrogen, Camarillo, CA, USA), 100 μg/ml penicillin and 100 μg/ml streptomycin (Invitrogen, Carlsbad, CA, USA). Cells were maintained in a humidified incubator at 37 °C in the presence of 5 % CO_2_.

### RNA extraction and qRT-PCR analysis

Total RNA from tissues and cells was extracted using Trizol reagent (TAKARA). RNA was reverse transcribed into cDNAs using the Primer-Script one step RT-PCR kit (TAKARA, Dalian, China). The cDNA template was amplified by real-time RT-PCR using the SYBR Premix Dimmer Eraser kit (TAKARA), which including U6 aliquot. Gene expression in each sample was normalized to GADPH or U6 expression. The primer sequences used were as follows: GAPDH, forward: 5’-GTCAACGGATTTGGTCTGTATT-3’ and reverse: 5’-AGTCTTCTGGGTGGCAGTGAT-3’; H19, forward: 5’-TTCAAAGCCTCCACGACTCT-3’ and reverse: 5’-: GCTCACACTCACGCACACTC-3’. FOXM1, forward: 5’-GAGACCTGTGATGGTGAGGC-3’ and reverse: 5’-ACCTTAACCTGTCGCTGCTC-3’. Real-time PCR reactions were performed using the ABI7500 system (Applied Biosystems, Carlsbad, CA, USA). The real-time PCRs were performed in triplicate. Relative expression fold change of mRNAs was calculated by the 2^−ΔΔCt^ method.

### Cell transfection

MiR-342-3p mimic or negative control mimic and has-miR-342-3p inhibitor or negative control inhibitor were purchased from Genepharma, Shanghai, China. The siRNAs specifically targeting H19 were synthesized by Genepharma, Shanghai, China. The siRNA sequences for H19 were si-H19-1, 5’-CCAACAUCAAAGACACCAUdTdT-3’, si-H19-2, 5’-UAAGUCAUUUGCACUGGUUdTdT-3’, and si-H19-3, sense 5’-CCCACAACA UGAAAGAAACTT-3’, and antisense: 5’-AUU UCU UUC AUG UUG UGG GTT-3’. SiRNAs specifically targeting FOXM1 were synthesized by Genepharma, Shanghai, China. The siRNA sequences for FOXM1 were si-FOXM1-1, 5’-CUCUUCUCCCUCAGAUAUA-3’, and si-FOXM-2, 5’-CCAGAGAGATGGACTGACA-3’. Transfections were performed using the Lipofectamine 2000 kit (Invitrogen) according to the manufacturer’s instructions.

#### Luciferase reporter assay

Human HEK293T cells (2.0 assays with a Molecular Imager) were co-transfected with 150 ng of either empty, pmir-GLO-NC, pmir-GLO-H19-wt or pmirGLO-H19-mut (Sangon biotech,China). Two ng of pRL-TK (Promega, Madison, WI, USA) were also co-transfected with miR-342-3p mimic or miRNA NC into HEK293T cells by using Lipofectamie 2000 (Invitrogen, USA). The relative luciferase activity was normalized to Renilla luciferase activity 48 h after transfection. Transfection was repeated in triplicate.

### RNA pull-down assay

To determine whether H19 is associated with the RISC complex, we performed RNA pull-down assay using biotin labeled H19 as a probe and then detected Ago2 from the pellet by western-blotting and miR-342-3p by qRT-PCR. The resultant plasmid DNA was linearized with restriction enzyme NotI. Biotin-labeled RNAs were in vitro transcribed with the Biotin RNA Labeling Mix (Roche Diagnostics, Indianapolis, IN, USA) and T7 RNA polymerase (Roche, Basel, Switzerland), treated with RNase-free DNase I (Roche) and purified with the RNeasy Mini Kit (Qiagen, Inc., Valencia, CA, USA). Cell extract (2 μg) was mixed with biotinylated RNA (100 pmol). Washed Streptavidin agarose beads (100 ml) were added to each binding reaction and further incubated at room temperature for 1 h. Beads were washed briefly three times and boiled in SDS buffer, and the retrieved protein was detected by standard western blot technique. The Ago2 antibodies used for pull-down were purchased from Abcam (Cambridge, MA, USA). The co-precipitated RNAs were detected by RT-PCR. Total RNAs and controls were also assayed to demonstrate that the detected signals were from RNAs specifically binding to Ago2.

### RNA immunoprecipitation (RIP)

RIP assay was performed as described previously [18], normal mouse IgG (Millipore) and SNRNP70 (Millipore) were used as negative control and positive control, respectively. The co-precipitated RNAs were detected by qRT-PCR. Total RNAs (input controls) and IgG were assayed simultaneously to demonstrate that the detected signals were the result of RNAs specifically binding to Ago2 (Cell signaling, USA).

### Immunohistochemistry (IHC)

Tumor specimens from nude mice were fixed in 4 % paraformaldehyde and then embedded in paraffin. Sections were used for the analysis of FOXM1 (1:100, Santa Cruz Biotechnology, Santa Cruz, CA). The samples were then incubated at 4 °C overnight with primary antibodies against FOXM1,and then treated with secondary antibody for 30 min and stained with diaminobenzidine (DAB) until brown granules appeared. Sections were blindly evaluated by two pathologists with light microscopy. Semi-quantitative of IHC was performed as described previously [30].

### Western-blotting

Western blot analysis to assess FOXM1 (1:500, Santa Cruz Biotechnology, Santa Cruz, CA) and GADPH (1:1000, Proteintech, China) expression was carried out as described previously [18]. GADPH primary antibody was purchased from Sigma (St. Louis, MO, USA). FOXM1 antibody was purchased from Santa Cruz Biotechnology, Inc. USA.

### Flow cytometric analysis

Cells transfected with desired plasmid or negative control were plated in six-well plates. After 48 h incubation, the cultures were incubated with propidium iodide for 30 min in the dark. Cultures were collected and analyzed for cell cycle using a flow cytometer (FACS Calibur, BD Biosciences, San Jose, CA, USA) after propidium iodide staining. Data were expressed as percentage distribution of cells in G0/G1, S and G2/M phases of the cell cycle.

### Cell invasion assays

For the invasion assays, 48 h after transfection, 2 × 10^5^ cells in serum-free media were placed into the upper chamber of an insert (8.0 μm, Millipore, Temecula, MA, USA) precoated with Matrigel (Sigma). The chambers were then incubated for 24 h in culture medium with 10 % FBS in the bottom chambers before examination. The cells on the upper surface were scraped and washed away, whereas the invaded cells on the lower surface were fixed and stained for 2 h. Finally, invaded cells were counted under a microscope and the relative number was calculated. Experiments were independently repeated three times.

### Xenograft mouse model

NOZ cells (1× 10^6^) stably expressing control shRNA or H19 shRNA, or miR-342-3p inhibitor (an anti-miR-342-3p Oligonucleotides, purchased from Hanbio, Shanghai, China) or H19 shRNA + miRNA-342-3p inhibitor were subcutaneously injected into either side of flank area of 3-week-old Male nude mice (n = 4 mice per group). Tumor volumes were measured (0.5 × length × width^2^) in mice on a weekly basis. After 4 weeks, mice were sacrificed, and tumors were excised and subjected to immune-histochemical analysis for FOXM1 expression. All animal experiments were performed in animal laboratory center of XinHua Hospital and in accordance with the Guide for the Care and Use of Laboratory Animals published by the US National Institutes of Health (NIH publication number 85–23, revised 1996). The study protocol was approved by the Animal Care and Use committee of Xinhua Hospital (approval ID: 2014041).

### Statistics analysis

All statistical analyses were performed using SPSS 13.0 (SPSS, Chicago, IL, USA). The expression differences between GBC and matched normal tissues were analyzed using paired samples *t*-test. Pearson’s coefficient correlation was used for expression correlation assay. The expression differences between the expression changes after transfection, S-phase fraction and invasion assay were analyzed using independent samples *t*-test. *P*-values were two-sided and a value of 0.05 was considered to be statistically significant.

## Results

### Identification of potential H19 targeting miRNAs

**Fig. 1:**
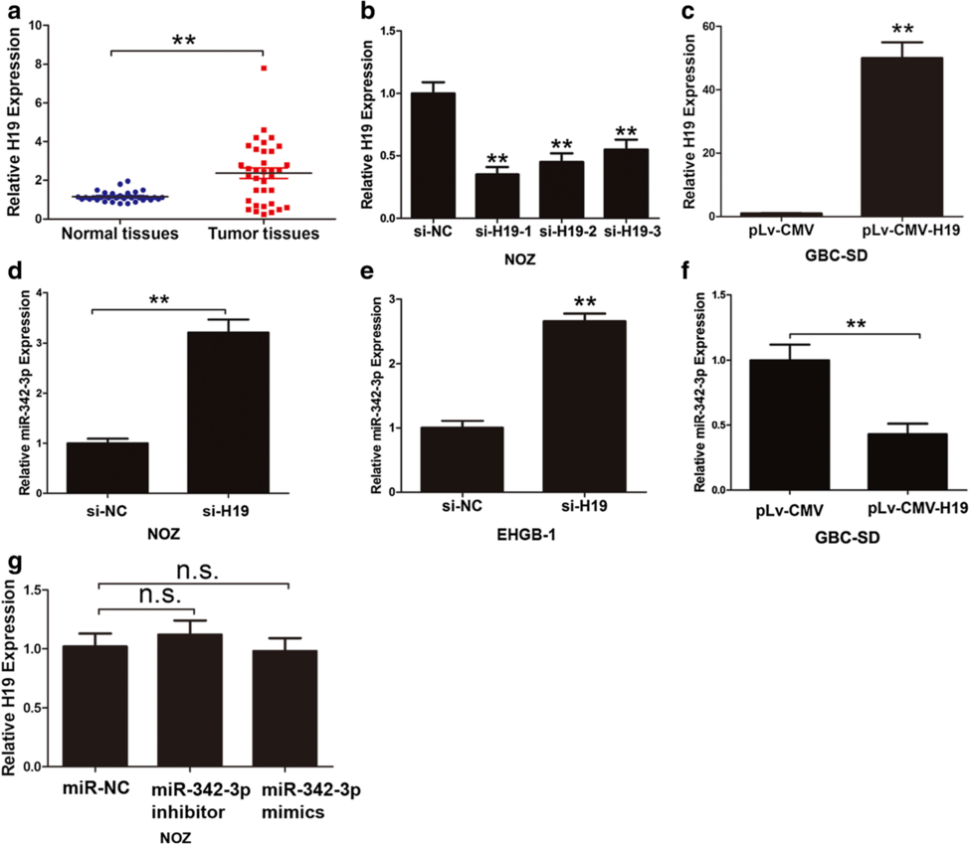
Expression of H19 in GBC and its effect on miR-342-3p. **a** Expression of H19 in 35 pair samples of GBC and adjacent normal tissues. The expression of H19 was normalized to GADPH, differences between GBC tissues and normal gallbladder tissues were compared by Paired Sample *T* test method (*n* = 35, *P* < 0.05). **b** H19 was knockdown by three siRNA targeting H19 (*P* < 0.05). **c** Expression level of H19 in GBC-SD cells after infection of lenti-virus vector which containing full-length of H19 was detected by qRT-PCR (*P* < 0.05). **d**–**e** The expression level of miR-342-3p after H19 silencing in NOZ and EHGB-1 cells was detected by qRT-PCR (*P* < 0.05). **f** The expression of miR-342-3p after over-expression of H19 was detected by qRT-PCR in GBC-SD cells (*P* < 0.05). **g** The expression of H19 after transfected of miR-342-3p inhibitor or mimic. All data were represented as the mean ± S.D. from three independent experiments, **P < 0.05

Previous study demonstrated H19 was overexpressed in GBC [29]. By expanding the cases of GBC specimens and paired normal tissues, we further confirmed that H19 was up-regulated in GBC tissues compared with adjacent normal tissues (Fig. 1a, *P* < 0.05). Based on previous study [29], H19 had the highest expression in NOZ cells measured in four GBC cell lines (SGC-996, GBC-SD, EHGB-1 and NOZ). NOZ cells was selected for H19 knockdown by three siRNA (Fig. 1a) and the efficient silencing of si-H19-1 was used in later experiments and GBC-SD cells were selected for over-expression experiment due to their relatively low H19 expression and easy-to-transfect feature (Fig. 1c).

A cohort of 20 potential miRNAs that could interact with H19 was predicted through starbase 2.0 and Miranda (http://starbase.sysu.edu.cn/ and http://www.microrna.org/) (Table 1). To search for specific target miRNA of H19 in GBC cells, H19 was knockdown in NOZ cells and then the miRNAs predicted above were measured by qRT-PCR in NOZ cells. The efficiency of interference of H19 in NOZ cells was confirmed by qRT-PCR (Fig. 1a). In Table 1, there were two miRNAs which were up-regulated more than 2.5-fold in response to H19 silencing. We focused on miR-342-3p, which had the greatest fold-change. MiR-342-3p expression levels were markedly increased after silencing H19 in NOZ and EHGB-1 cells (*P* < 0.05) (Fig. 1d–e). On the contrary, miR-342-3p expression was decreased significantly after over-expression of H19 in GBC-SD cells (*P* < 0.05) (Fig. 1c and f). However, the exogenous miR-342-3p inhibitors or mimics did not alter the expression of H19 (Fig. 1g).

**Table 1:** QRT-PCR verified the fold change expression of predicted miRNAs in NOZ cells after H19 knockdown

miRNAs	fold change
hsa-miR-194-5p	2.68
hsa-miR-148a-3p	1.78
hsa-miR-148b-3p	1.43
hsa-miR-103a-3p	1.34
hsa-miR-93-5p	1.56
hsa-miR-107	1.21
hsa-miR-454-3p	1.52
hsa-miR-491-5p	1.89
hsa-miR-138-5p	0.98
hsa-miR-140-5p	1.55
hsa-miR-339-5p	1.44
hsa-miR-193b-3p	1.52
hsa-miR-152-3p	1.38
has-miR-342-3p	3.32
hsa-miR-130b-3p	1.12
hsa-miR-130a-3p	1.46
hsa-miR-370-3p	1.01
has-miR-19b	0.88
hsa-miR-519d-3p	2.01
hsa-miR-216b-5p	1.58

### The potential mechanism of the negative regulation of miR-342-3p by H19

**Fig. 2:**
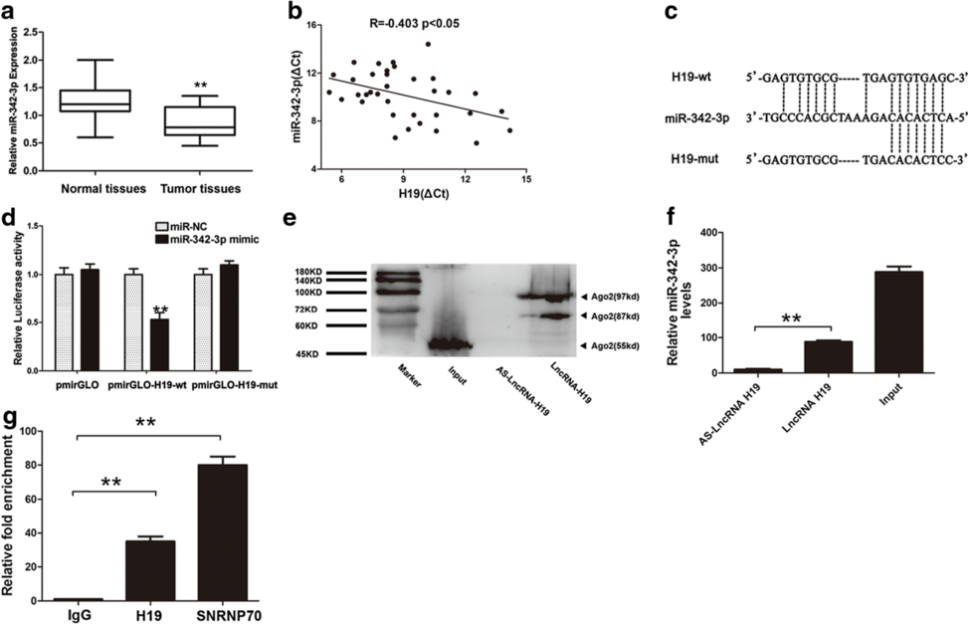
MiR-342-3p was a target of H19. **a** Expression level of miR-342-3p in pair samples of GBC and normal tissues was detected by qRT-PCR (*n* = 35, *P* < 0.05). The expression of miR-342-3p was normalized to U6. **b** Pearson’s correlation was used for correlation analysis between the expression of H19 mRNA and miR-342-3p mRNA (*R* = −0.403, *P* < 0.05). **c** Representation the H19 and miR-342-3p binding site by miRanda (http://www.microrna.org/). **d** Luciferase activity in HEKT293 cells cotransfected with miR-342-3p mimic and luciferase reporters containing control vector, pmir-Glo-H19-wt and pmir-Glo-H19-mut (*P* < 0.05). **e** Pull-down assay was conducted using Biotin-labeled H19 probe and detected the AGO2 expression by western-blotting assays. Antisense of the H19 probe was used as negative control. **f** Pull-down assay was conducted using Biotin-labeled H19 probe and miR-342-3p level was detected in the substrate of pull-down assay by qRT-PCR, antisense of the H19 probe was used as negative control. **g** H19 mRNA level was detected in the substrate of RIP assay by qRT-PCR. All data were represented as the mean ± S.D. from three independent experiments, **P < 0.05

Furthermore, we detected that miR-342-3p was down-regulated in GBC tissues compared to adjacent normal tissues (*P* < 0.05), the expression of miR-342-3p was normalized to U6 (Fig. 2a). In addition, the expression level of H19 was significant negatively correlation with miR-342-3p (*R* = −0.403, *P* < 0.05) (Fig. 2b). These results indicated that miR-342-3p might have an interaction with H19.

To explore whether miR-342-3p was targeted and directly bound to H19, fragments of wild-type and mutated H19 cDNA sequence containing the putative miR-342-3p recognition site (predicted in starbase 2.0) (Fig. 2c) was cloned. Dual reporter luciferase was performed in HEK293T cells. Dual luciferase reporter assay results indicated that miR-342-3p mimic significantly decreased the luciferase activities of pmir-Glo-H19-wt (49 %) but not pmir-Glo-H19-mut (Fig. 2d).

MiRNAs and siRNAs guided Argonaute proteins (Ago proteins including Ago2) to silence mRNA expression through RNA-Induced Silencing Complex (RISC) [31–33]. Then we conducted pull-down assay to explore whether H19 functioned through RISC complex via interaction with Ago2. We showed that H19 probe harbored the Ago2 protein and detected the expression of miR-342-3p in the same pellet, the antisense strand of H19 probe was used as negative control (Fig. 2e and f). We also demonstrated that H19 was preferentially enriched in Ago2-containing beads compared with the beads harboring control immunoglobulin G (IgG) antibody. U1 small nuclear ribonucleoprotein 70 kDa (SNRNP70), a gene coding SNRNP70 protein associated with U1 spliceosomal RNA [34] was used as positive control (Fig. 2g). These results suggested that H19 directly targeted miR-342-3p.

### H19 modulated expression of endogenous miR-342-3p targets FOXM1

**Fig. 3:**
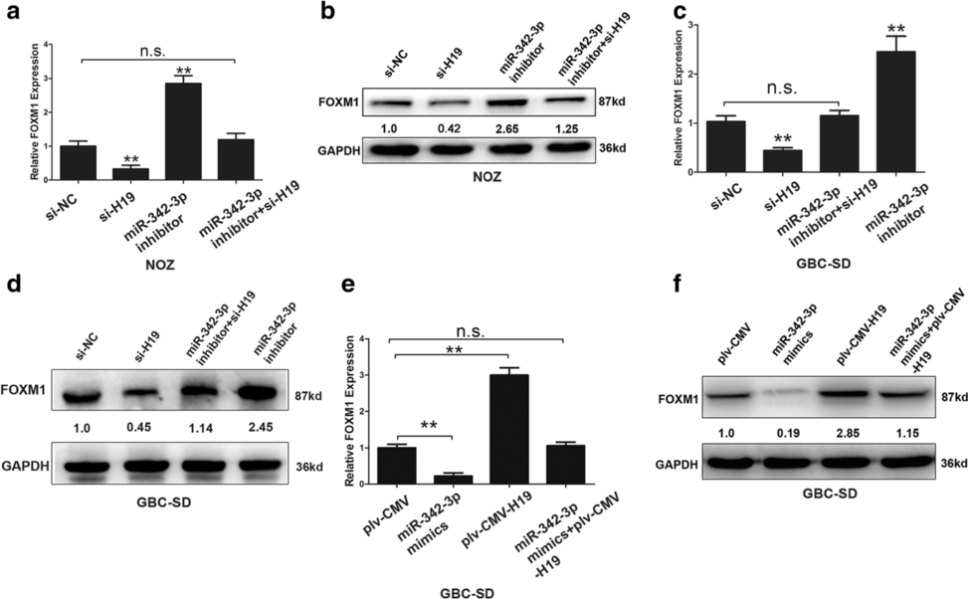
H19 modulated expression of endogenous miR-342-3p targeting FOXM1. **a**-**b** The mRNA levels and protein levels of FOXM1 in NOZ cells transfected with si-NC, si-H19, miR-342-3p inhibitor, and si-H19 + miR-342-3p inhibitor. **c**-**d** The mRNA levels and protein levels of FOXM1 in GBC-SD cells transfected with si-NC, si-H19, si-H19 + miR-342-3p inhibitor, and miR-342-3p inhibitor. **e**-**f** The mRNA levels and protein levels of FOXM1 in GBC-SD cells transfected with pLv-CMV, miR-342-3p mimic, pLv-CMV-H19, miR-342-3p mimic + pLv-CMV-H19. All data were represented as the mean ± S.D. from three independent experiments, **P < 0.05

FOXM1 was a well-accepted oncogene that is targeted by miR-342-3p in cervical cancer [35]. We next verified whether H19 modulated the expression of FOXM1 by targeting miR-342-3p in GBC cells. Compared to the control group, the RNA and protein levels of FOXM1 were down-regulated after H19 silencing in NOZ and GBC-SD cells and up-regulated by transfecting with miR-342-3p inhibitors; but in the group of co-transfecting with miR-342-3p inhibitor and siRNA-H19, the modulating effects of H19 on FOXM1 were diminished (Fig. 3a–d). Similarly, the RNA and protein levels of FOXM1 were both increased after cells transfected by overexpression H19 in GBC-SD, however, the effects were counteracting after co-transfection of H19 plasmids and miR-342-3p mimic (Fig. 3e and f).

### FOXM1 promoted cell invasion and cell cycle in GBC cells

**Fig. 4:**
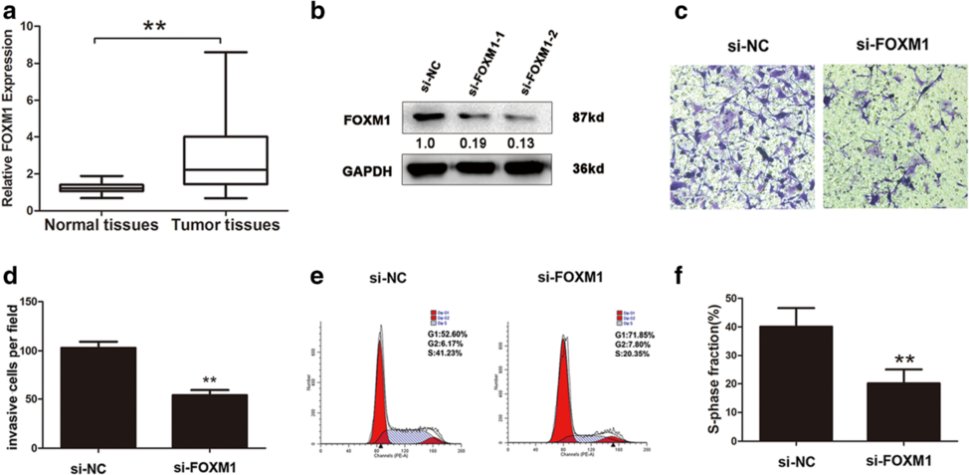
FOXM1 promoted cell invasion and cell cycle in GBC cells. **a** RNA expression level of FOXM1 and normal tissues was detected by qRT-PCR in GBC tissues (*n* = 35, *P* < 0.05). The expression of FOXM1 was normalized to GADPH. **b** The expression of FOXM1 after knockdown was detected in NOZ cell by western-blotting assays. **c**-**d** cells invasion ability and invasive cells number were detected after FOXM1 knockdown in NOZ cell (*P* < 0.05). **e**-**f** Cell cycle was analyzed after knockdown of FOXM1 in NOZ cell. All data were represented as the mean ± S.D. from three independent experiments, **P < 0.05

To investigate the functional effects of FOXM1 on GBC cells, we measured the RNA levels of FOXM1 in GBC tissues and their matched normal tissues via qRT-PCR. We found that FOXM1 was up-regulated in GBC tissues (Fig. 4a). After FOXM1 silencing, the number of invasive cells was markedly decreased compared to the control group (Fig. 4b–d). Cell cycle analysis indicated that NOZ cells were arrested in G0/G1-phase and the cells number in S-phase was significantly decreased after FOXM1 knockdown (Fig. 4e and f).

### H19/miR-342-3p/FOXM1 axis on cell invasion and cell cycle in GBC cells

**Fig. 5:**
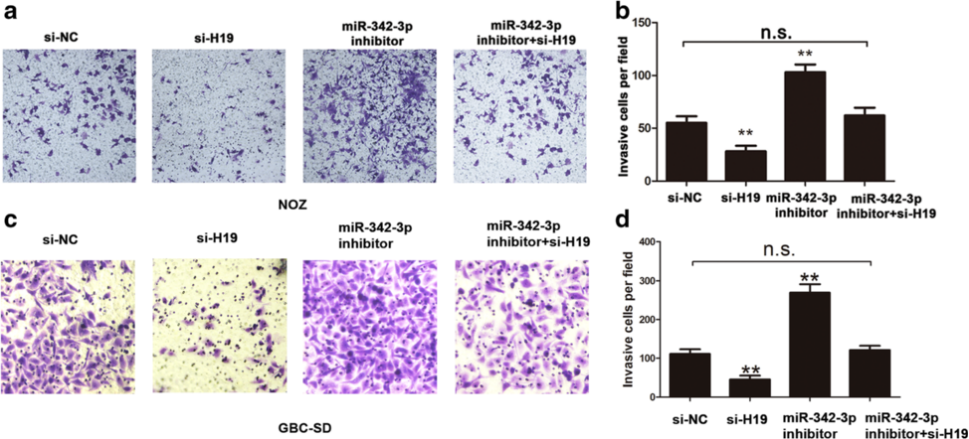
H19/miR-342-3p/FOXM1 axis on cell invasion in GBC cells. **a**-**b** Cell invasion ability and invasive cells number were detected by transfecting si-NC, siH19, miR-342-3p inhibitor, and siH19+ miR-342-3p inhibitor into NOZ cells (*P* < 0.05). **c**-**d** Cell invasion ability and invasive cells number were detected by transfecting si-NC, siH19, miR-342-3p inhibitor, and siH19+ miR-342-3p inhibitor into GBC-SD cells (*P* < 0.05). All data were represented as the mean ± S.D. from three independent experiments, **P < 0.05

**Fig. 6:**
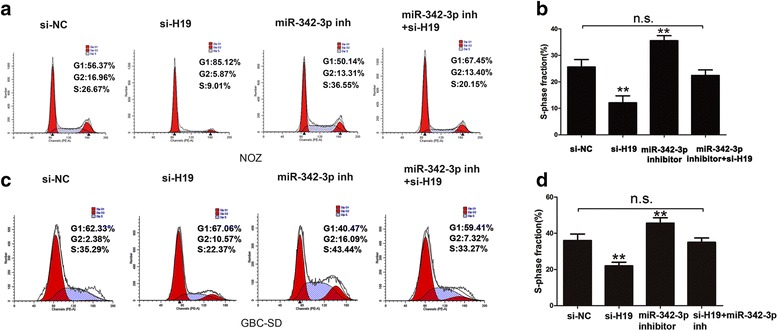
H19/miR-342-3p/FOXM1 axis on cell cycle in GBC cells. **a**-**b** cell cycle were detected by transfecting si-NC, siH19, miR-342-3p inhibitor and siH19+ miR-342-3p inhibitor into NOZ cells (*P* < 0.05). **c**-**d** Cell cycle was detected by transfecting si-NC, siH19, miR-342-3p inhibitor and siH19+ miR-342-3p inhibitor into GBC-SD cells (*P* < 0.05). All data were represented as the mean ± S.D. from three independent experiments, **P < 0.05

We continued to explore the effects of H19/miR-342-3p/FOXM1 axis on invasion and cell cycle in GBC cells. Initially, compared to the control group, knockdown of H19 decreased the number of invasive cells in NOZ and GBC-SD cells, but was reversed by co-transfection of siRNA-H19 and miR-342-3p inhibitor simultaneously (Fig. 5a–d). In cell cycle assay in NOZ and GBC-SD cells, knockdown of H19 induced cell cycle arrest in G0/G1 phase, but was reversed by co-transfection of siRNA-H19 and miR-342-3p inhibitor (Fig. 6a–d).

### H19 oncogenic activity is in part through negative regulation of miR-342-3p in vivo

**Fig. 7:**
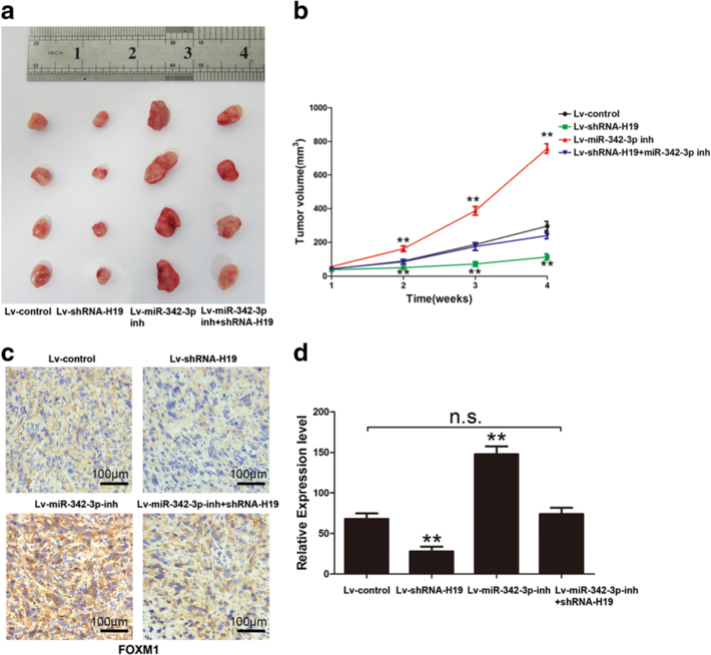
H19 oncogenic activity is in part through negative regulation of miR-342-3p in vivo (**a**) Representation picture of tumor formation of xonograft in nude mice in lv-control, lv-shRNA-H19, lv-miR-342-3p inhibitor and lv-shRNA-H19+ miR-342-3p inhibitor group, respectively (each group *n* = 4). **b** Summery of tumor volume of mice which were measured in every week in lv-control, lv-shRNA-H19, lv-miR-342-3p inhibitor and lv-shRNA-H19+ miR-342-3p inhibitor group, respectively. **c** Representation of FOXM1 expression in xenografts tumor using IHC method in lv-control, lv-shRNA-H19, lv-miR-342-3p inhibitor and lv-shRNA-H19+ miR-342-3p inhbitor group, respectively. **d** Statistical analyses of IHC results in xenograft tumor in lv-control, lv-shRNA-H19, lv-miR-342-3p inhbitor and lv-shRNA-H19+ miR-342-3p inhbitor group, respectively

To verify our in vitro findings, we established an in vivo xenograft model in nude mice. Four lenti-virus constructed cells including lv-control, lv-sh-H19, lv-miR-342-3p inh and lv-miR-342-3p inh + lv-sh-H19 were well cultured and were injected subcutaneously. Tumors were allowed to form and grow for 4 weeks (*n* = 4 for each group). Tumor volumes were measured weekly. At the end of the fourth weeks, the mice were sacrificed and tumors excised, we found that H19 knockdown significantly reduced the tumor volume of NOZ cells when compared with control group. Lv-miR-342-3p inhibitor significantly increased the oncogenic ability of NOZ cells. Simultaneous knockdown of H19 and inhibited miR-342-3p had no impact on tumor formation (Fig. 7a and b). Immuno-histochemical (IHC) was used to detect the expression of FOXM1 in xenograft tumor. In consistent with in vitro results, knockdown of H19 markedly decreased the protein expression level of FOXM1, and inhibiting miR-342-3p significantly up-regulated FOXM1 expression (Fig. 7c). Semi-quantitative of IHC was also summarized in Fig. 7d.

**Fig. 8:**
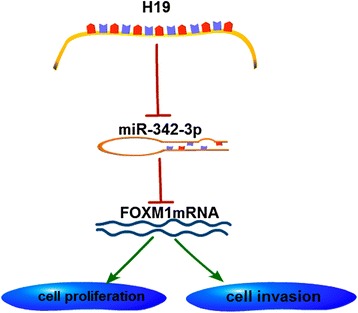
Model for H19-regulating FOXM1 by acting as a ceRNA

To sum up, a ceRNA model was proposed to summarize H19/miR-342-3p/FOXM1 pathway (Fig. 8). H19 negatively regulated miR-342-3p in GBC, through binding to miR-342-3p directly. In addition, we found that miR-342-3p targeted FOXM1 and negatively regulated FOXM1 expression, indicating that H19 could influence the expression of FOXM1 in GBC through miR-342-3p (Fig. 8).

## Discussion

In recently reports, H19 exerted two opposite effects depending on tumor types: it functioned as an oncogene in ovarian cancer [36], colorectal cancer [27], breast cancer [37] and glioblastoma [38]; whereas, it exerted tumor suppression effects in hepatocellular carcinoma [39], prostate cancer [40] and Wilms’ tumor [41]. In our previous study, we found that H19 functions as an oncogene in GBC and promoted cell proliferation by upregulating the AKT2 expression level in GBC cells, and upregulation of H19 promoted the epithelial-mesenchymal transition (EMT) [28, 29]. Based on these previous researches, our further revealed that H19/miR-343-5p/FOXM1 might be a potential ceRNA regulatory network in GBC.

To uncover the mechanism of H19 regulation in GBC, we discovered that H19 shared miR-342-3p response element with FOXM1, an important oncogene that has been reported to be associated with many cancers [42–44]. Interestingly, miR-342-3p exerted tumor suppression effects in GBC, which was consistent with other findings in lung cancer [45], hepatocellular carcinoma [46] and cervical cancer [35]. Furthermore, the results of dual-luciferase reporter assay, RNA-binding protein immunoprecipitation assay and RNA pull-down assay indicated that H19 was a target of miR-342-3p.

FOXM1 was known to regulate a transcriptional program required for mitotic progression [47]. Numerous articles have reviewed that FOXM1 function as oncogene and could be a therapeutic target in many kinds of cancer [48, 49]. Our results indicated that down-regulation of FOXM1 inhibited cell invasion in GBC cells, which was consistent with others reports [50]. We further defined that down-regulating FOXM1 arrested GBC cells in G0/G1 phase also in accordance with reports showing that FOXM1 promotes mitotic gene expression [51]. Previous studies about transcription regulation signaling pathway suggested that FOXM1 transcriptionally regulated of downstream genes such as TGF-β/SMAD3/4 to promote cancer metastasis [52] and induced PRX3 to regulates stemness and survival of colon cancer cells [53]. For FOXM1 downstream study, a microarray analysis indicated that LncRNA-FRLnc1 was regulated by FOXM1 [54]. However, regulation by up-stream factors that activated FOXM1 has remained enigmatic. Our findings indicate that either in vitro or in vivo, H19 up-regulation is sufficient to up-regulate FOXM1 and promotes GBC proliferation.

It is worth noting that the relationship between H19 and FOXM1 depends on miR-342-3p. Our results proposed that H19 functioned as a ceRNA for miR-342-3p, a new miRNA, to regulate FOXM1 epigenetically. Previous reports about H19 in post-transcriptional regulation included that placental specific expression of miR-675 was encoded by H19 at gestational time point to target insulin-like growth factor 1 receptor [55]. Moreover, another reports supported that H19-derived miR-675 targeting TGF-β signaling [40, 56] and tumor suppressor RB [57]. We discovered that knocked down H19 in GBC cell lines increased the endogenous level of miR-342-3p (Fig. 1d, e), and these effects were neutralized by exogenous miR-342-3p inhibitor (Fig. 3a–d). The expression of H19 was not changed by miR-342-3p mimic or miR-342-3p inhibitor, which indicated that H19 was the upstream of miR-342-3p. Our study suggests a potential regulation pathway that involving H19 in both molecular and biological aspects in GBC.

## Conclusions

In conclusion, we suggest a potential ceRNA regulatory network involving H19 and miR-342-3p in the modulation of FOXM1 expression. This mechanism may contribute to a better understanding of GBC pathogenesis and provides new therapeutic target as well as prognostic marker in GBC.

## Abbreviations

ceRNA: Competing endogenous RNA
FOXM1: Forkhead box M1
GAPDH: Glyceraldehyde-3-phosphate dehydrogenase
GBC: Gallbladder cancer
IHC: Immunohistochemistry
lncRNA: Long non-coding RNA
PCR: Polymerase chain reaction
RIP: RNA immunoprecipitation
