# Correction to: Long non-coding RNA H19 regulates FOXM1 expression by competitively binding endogenous miR-342-3p in gallbladder cancer

**DOI:** 10.1186/s13046-022-02277-6

**Published:** 2022-02-09

**Authors:** Shou-Hua Wang, Fei Ma, Zhao-hui Tang, Xiao-Cai Wu, Qiang Cai, Ming-Di Zhang, Ming-Zhe Weng, Di Zhou, Jian-Dong Wang, Zhi-Wei Quan

**Affiliations:** 1 Department of General Surgery, Xinhua Hospital, Shanghai Jiao tong University School of Medicine, 1665 Kong Jiang Road, Shanghai, 200000 China; 2 Department of Oncology, Xinhua Hospital Affiliated to Shanghai Jiao tong University School of Medicine, Shanghai, 200092 China

**Correction to: J Exp Clin Cancer Res 35, 109 (2016)**

**https://doi.org/10.1186/s13046-016-0436-6**

Following publication of the original article [1], the authors identified a minor error in Fig. 5; specifically:

- In Fig. 5a, an incorrect image was used for the si-NC group; the image has been replaced by the correct image.

The corrected figure is provided here. The correction does not have any effect on the results or conclusions of the paper.

**Fig. 5:**
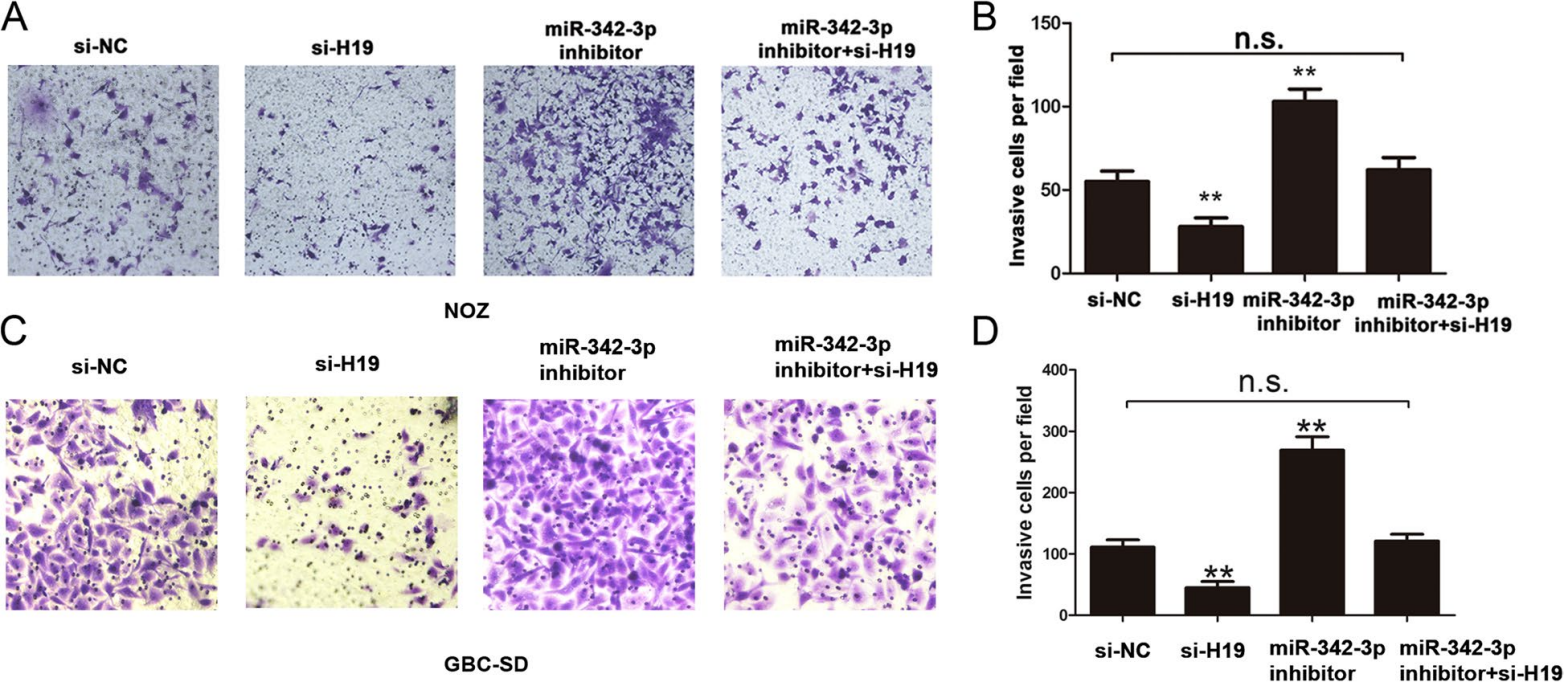
H19/miR-342-3p/FOXM1 axis on cell invasion in GBC cells. **a-b** Cell invasion ability and invasive cells number were detected by transfecting si-NC, siH19, miR-342-3p inhibitor, and siH19+ miR-342-3p inhibitor into NOZ cells (*P* < 0.05). **c-d** Cell invasion ability and invasive cells number were detected by transfecting si-NC, siH19, miR-342-3p inhibitor, and siH19+ miR-342-3p inhibitor into GBC-SD cells (*P* < 0.05). All data were represented as the mean ± S.D. from three independent experiments, **P < 0.05
